# Semiconductor Nanomaterials-Based Fluorescence Spectroscopic and Matrix-Assisted Laser Desorption/Ionization (MALDI) Mass Spectrometric Approaches to Proteome Analysis

**DOI:** 10.3390/ma6125763

**Published:** 2013-12-09

**Authors:** Suresh Kumar Kailasa, Kuang-Hung Cheng, Hui-Fen Wu

**Affiliations:** 1 Department of Chemistry, S. V. National Institute of Technology, Surat 395007, India; E-Mail: sureshkumarchem@gmail.com; 2 Institute of Biomedical Sciences, National Sun Yat-Sen University, Kaohsiung 804, Taiwan; E-Mail: khcheng@faculty.nsysu.edu.tw; 3 Department of Chemistry, National Sun Yat-Sen University, Kaohsiung 804, Taiwan; 4 Center for Nanoscience and Nanotechnology, National Sun Yat-Sen University, Kaohsiung 804, Taiwan; 5 Doctoral Degree Program in Marine Biotechnology, National Sun Yat-Sen University, Kaohsiung 804, Taiwan; 6 School of Pharmacy, College of Pharmacy, Kaohsiung Medical University, Kaohsiung 806, Taiwan

**Keywords:** quantum dots, fluorescent and affinity probes, biomolecules, fluorescence spectroscopy, MALDI-MS

## Abstract

Semiconductor quantum dots (QDs) or nanoparticles (NPs) exhibit very unusual physico-chemcial and optical properties. This review article introduces the applications of semiconductor nanomaterials (NMs) in fluorescence spectroscopy and matrix-assisted laser desorption/ionization mass spectrometry (MALDI-MS) for biomolecule analysis. Due to their unique physico-chemical and optical properties, semiconductors NMs have created many new platforms for investigating biomolecular structures and information in modern biology. These semiconductor NMs served as effective fluorescent probes for sensing proteins and cells and acted as affinity or concentrating probes for enriching peptides, proteins and bacteria proteins prior to MALDI-MS analysis.

## 1. Introduction

Nanomaterials have been integrated in various analytical tools to gain prominence advances in bioanalytical and proteomics research communities since last decade. Among many different nanomaterials, the colloidal semiconductor nanocrystals (NCs) or QDs have been demonstrated as potential optical probes in bioanalytical chemistry due to their special physico-chemical and optical properties including size-tunable emission, continuous absorption profile to blue of the band edge and stability against photobleaching which allows a novel and often counter-intuitive applications in bioanalytical research [1,2,3,4,5]. Although several materials exhibit semiconducting behavior, silicon has been most widely used than any other semiconductors. Silicon is a group IV and its outer orbital of an individual atom is only half filled. After in-depth studies on the structural properties of nanomaterials, the colloidal nanocrystalline semiconductors are collectively referred as the comprising elements from the periodic groups II–VI, III–V or IV–VI, are roughly spherical and with sizes typically in the range 1–20 nm in diameter. Over the last two decades, the development of semiconductor nanomaterials has received tremendous progress in their preparation as well as in understanding their optical and electronic properties [6,7,8]. Indeed, the QDs exhibited tremendous applications in biodiagnostics, bioimaging, photonics, optoelectronics, and sensors [9]. Importantly, different sizes of QDs can be excited simultaneously by a single wavelength and emit with distinctly different colors. In this connection, the swift progress of this field of research has been well addressed in monographs and review articles dealing with the synthesis, physico-chemical, optical properties and applications of QDs [10,11,12]. These reports reveal that the semiconductor nanomaterials are attractive and have interesting structures for proteome analysis with high sensitivity. Therefore, we briefly introduced on semiconductor nanomaterials and their uses as the optical probes in fluorescence spectroscopy and as affinity probes in MALDI-MS for proteome analysis.

## 2. Overview of Colloidal Semiconductor QDs

In 1959, R. Feynman presented a perspective lecture at Caltech, and suggested that “strange phenomena” would occur when a material’s size is reduced to nanometer scale [13]. This can cause radical changes in semiconductor nanomaterials when quantum-size phenomena (quantum confinement) take over conventional bulk properties. The semiconductor NCs or QDs are in fact artificial atoms. The smallest QDs (<1 nm) are made of ~100 atoms while the largest QDs (>20 nm) can be made from 100,000 atoms. It is confirmed that semiconductor NCs or QDs in which excitons are confined in all three spatial dimensions. This confinement can be realized by fabricating the semiconductor in very small size, typically several hundred to thousands of atoms per particle. The most striking unique property is that the absorption and emission of QDs can be “tuned” to any chosen wavelength by simply changing their size. The size of the structure limits the exciton-Bohr radius of the bound electron-hole pairs, leading to altered electronic and optical properties [14,15]. This is because their unique size and shape-dependent optical properties are entirely different at the atomic/molecular level. Due to their discrete energy levels in both valence band and conduction band, a limited number of atoms may be present in each particle. Therefore, the absorbed energy is greater than the band gap energy, electrons (represented by the solid circle) in the valence band could absorb the energy and “jump” to the conduction band, forming short-lived electron-hole pairs and the bound state of the electron–hole pair is called an “exciton” [16]. In the quantum mechanics, this “hole” is assumed to behave as a “particle” with a certain effective mass and positive charges. Then, the electrons and holes (represented by the open circle) can quickly recombine, and photons are emitted with a specific energy corresponding to the band gap, which is the band edge emission. Therefore, charge carriers have to assume higher kinetic energies, which lead to increase in band gap and quantization of the energy levels to discrete values. This entire phenomenon is “quantum confinement effect” [17] and the theory is that behavior of exciton ensembles more like quantum mechanics of atom in 3-D model of a particle in a box [18]. Indeed, the Bohr exciton radii in bulk semiconductor QDs are approximately 3–5 nm; however, in unusual situations, the exciton size can be exceeded in the crystal dimensions by reducing particle size to a few nm. In nanometer range (1–20 nm), the physical properties of nanomaterials become very sensitive to the shape and size. The quantum size effect occurs when the size of the nanocrystal becomes comparable to or smaller than the natural length of electrons and holes. To define precisely, the Bohr radius can be utilized as a convenient length scale and the Bohr radius of a particle is given by the following equation:

(1) $$a_{B}=\;\varepsilon \frac{m}{m^{*}}a_{0}\;$$

where ε is the dielectric constant of the material; $m^{*}$ is the reduced mass of the particle; m is the rest mass of the electron, and $a_{0}$ is the Bohr radius of the hydrogen atom [19].

### Types of Semiconductor QDs

To demonstrate the related theoretical framework, we discuss the theoretical understanding of energy states for conduction electrons and their distribution related to shape and size of the materials and they are classified as the following [20]. 

**Three dimensional system (3D)**: In bulk materials, carriers can freely move in all three dimensions and the material is referred as a three dimensional (3D) system. In this system, energy of the conduction electron equation is already well defined [21,22]. Similarly, quantum well can be defined that if one of the spatial dimensions of the material is in nanometer range, while the other two remain larger than the resulting structure. Quantum well is referred as a two-dimensional (2D) system since carriers are free to move in two dimensions and confined in one dimension. This 2D energy of conduction electrons is represented by the following equation.

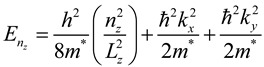
(2)
where *n_z_* is the sub-band index. The carriers are confined in the z-dimension with thickness *L_z_* and free in *x-* and *y-* dimensions.

The two spatial dimensions of a material are in nanometer range but the other one is larger than the resulting structure is referred as quantum wires. It is a one-dimensional (1D) system. Here, carriers are free moving in one dimension and are confined in the other two dimensions. Hence, the energy of the conduction electrons can be defined as:

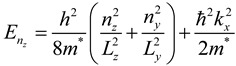
(3)
where *L_y_* and *L_z_* are lengths of the rectangular wire in *x-*, * y-* and *z-* dimensions. In this system, the energy levels depend on two quantum numbers *n_y_* and *n_z_*, respectively. 

**Figure 1:**
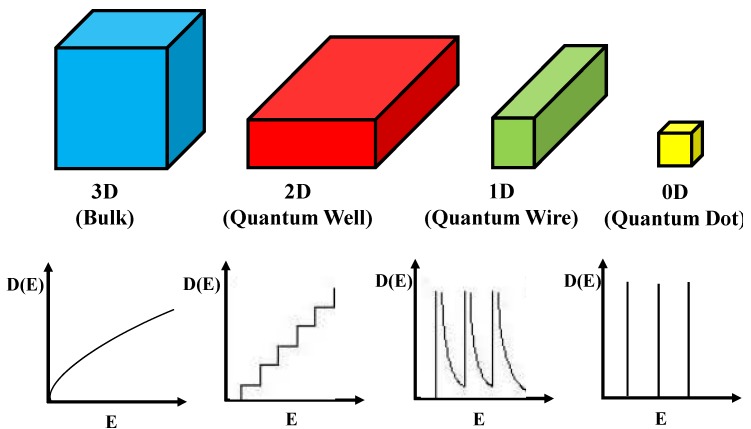
Schematic diagram of density of states for 3D, 2D, 1D and 0D systems.

Similarly, quantum dot can be defined as three dimension materials are in nanometer range and it is considered to be a zero-dimensional (0D) system since carriers are confined in all three dimensions. In this system, energy levels are entirely discrete and written as:

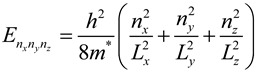
(4)
where *L_x_*, *L_y_* and *L_z_* are the dimensions in *x-*, * y-*, and *z-* dimensions. The energy states depend on three quantum numbers *n_x_*, * n_y_* and *n_z_*, respectively. The schematic diagram of density of states for 3D, 2D, 1D and 0D was shown in Figure 1.

Furthermore, the confinement energy (the difference between the gap for low dimensional systems and 3D systems) is proportional to the size of the system. It is noticed that effective masses of charge carriers in semiconductors are less than the free electron mass, so that quantum confinement energy is observed at room temperature comparatively at a larger size (of few nanometers depending on the material) in contrast to the case of a metal, where quantum confinement energy can be observed at a low temperature only for a cluster of few atoms. Due to confinement of charge carriers, the discritization of energy levels and widening of energy gap can be realized by optical absorption and photoluminescence measurements. As a consequence of these structures, semiconductor nanomaterials were demonstrated several altered characteristics compared to their bulk constituents. These altered physical, electronic and optical properties of semiconductor nanomaterials play key role in the development of analytical method for the analysis of proteins by fluorescence spectroscopy and MALDI-MS.

## 3. Semiconductor QDs-Based Fluorescence Approaches for Biomolecules Assays

One of the fundamental goals in modern proteomics is to understand the complex spatio–temporal interplay of proteins from the cellular to the integrative level. To study these interactions, the integration of unique structural characters of semiconductor QDs or NCs with analytical instruments have created many new-style tools for proteome analyses. Since, QDs have attracted broad attention to use them as optical probes for fluorescence based approaches in proteomics, which is due to their unique optical and electronic properties. Compared to organic dyes, QDs or NCs shows high quantum yield, high molar extinction coefficients (~10–100 times), broad absorption with narrow, symmetric photoluminescence spectra (full-width at half-maximum ~25–40 nm) spanning the UV to near-infrared [23]. It should be noticed that the dependence of optical properties of QDs or NCs is mainly dependent on particle size and the internal structure. It is well known that the number of surface atoms increases when the crystal size is much smaller, which can also impact the optical properties. The atoms on the surface of a crystal facet are incompletely bonded within the crystal lattice, thus disrupting the crystalline periodicity and leaving one or more “dangling orbital” on each atom pointed outward from the crystal [24]. Generally, QDs are highly faceted, and each surface contains a periodic array of unpassivated orbitals with two-dimensional translational symmetry, which may form a band structure similar to that of three-dimensional crystal itself [25,26]. If these surface energy states are within the semiconductor band gap, they can trap charge carriers at surface, thereby reducing overlap in between electron and hole, increasing the probability of nonradiative decay events. Due to their wide optical tenability, QDs have attracted broad attention in multidisciplinary science. QDs are elongated structures and emitted linearly polarized light with a wide energy separation between the absorption and emission maxima (Stokes shift). Quantum wells are well-established components of optoelectronic devices, and their colloidal “disk” analogues have recently been described, which may have novel piezoelectric applications (Reprinted with permission from Ref. [24]). These unique optical properties make them efficient optical candidates to probe the trace level biomolecules in complex samples without any sample pretreatment.

### 3.1. Semiconductor QDs-Based FRET and BRET Approaches

The luminescence of semiconductor QDs is very sensitive to their surface states. The fluorescence transduction is based on the principle of physico-chemical interactions between semiconductor QDs and analytes. These interactions play a key role in the efficiency of the radiative recombination, either leading to photoluminescence activation or quenching [27]. These changes can be generated by the direct interaction between the analyte and the semiconductor QDs surface, which allows the selective detection of a multitude of compounds. These mechanisms are based on energy flow (transfer of electronic excitation energy) between the components of nanoassemblies. This can occur when light energy absorbed by QDs (donor) is transferred to a nearby acceptor species, such as an organic fluorophore (acceptor) in a process called Förster (Fluorescence) Resonance Energy Transfer (FRET) [28]. The Förster radius (*R*_0_) can be written in Equations (5) and (6).

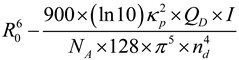
(5)

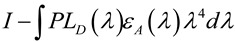
(6)
where *к^2^_p_* = 2/3 for randomly oriented dipoles; *Q_D_* is the quantum dot efficiency of the donor; *N_A_* is the Avagadro’s number; *n_d_* is the reflective index of the solution; *I* is the spectral overlap between the donor emission and acceptor absorption and further expressed in Equation (6), in which *PL_D_*(*λ*) is the normalized emission intensity of the donor QDs, *ε_A_*(*λ*) is the absorption extinction coefficient of the acceptor and *n_d_* is the value for water (1.333) at 580 mm. It should be noted that the rate of energy transfer depends on the distance between the donor and the acceptor, their relative orientations and the spectral overlap. Similarly, Bioluminescence Resonance Energy Transfer (BRET) is ideally suited for luminescent QDs since it avoids the difficulties in using QDs as acceptor fluorophores. BRET mechanism deals with the photon generating process to transfer the excitation energy nonradiatively to a proximal fluorescent acceptor through chemical reaction. Therefore, we will provide an overview of the literature dealing with the different semiconductor QDs-based fluorescent probes for the analysis of biomolecules by fluorescence spectroscopy.

**Figure 2:**
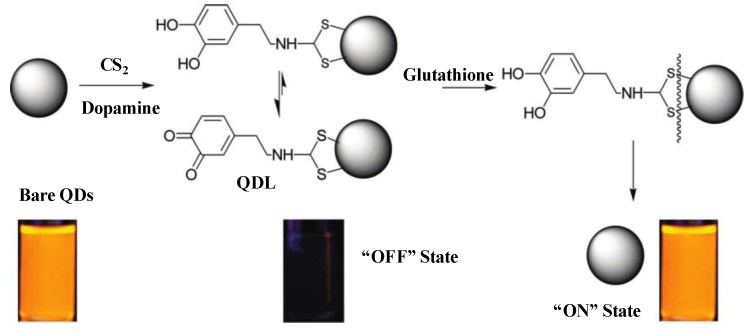
Schematic representation of quantum dots (QDs)-based fluorescence ON/OFF mechanism for the detection of glutathione. Reprinted with permission from [30].

Fluorescence “ON” and “OFF” mechanism allows us to detect biomolecules with high sensitivity and resolution. Since the resolution can be measured based on the switching of the fluorescent probes between two distinctively different fluorescent states, either fluorescent “ON” and “OFF” states or two fluorescent states with distinct color. Furthermore, the fluorescent probes must be actively varied (usually via photoswitching or photoactivation) in time to ensure that only an optically resolvable subset of fluorophores is activated at any time in a diffraction—limited region, thereby allowing their localization with high accuracy [29]. For example, Santra’s group described a method for synthesis of dopamine dithiocarbamate (DDTC) capped with CdS:Mn/ZnS core/shell QDs using a water-in-oil (W/O) microemulson mechanism [30]. These QDs acted as fluorescent probes to detect glutathione (GSH) through fluorescence ON/OFF mechanism (Figure 2). In this method, photoluminescence (PL) intensity of bare QDs was greatly quenched by the addition of DDTC, which is due to annihilation pathway by the addition of dopamine. This system was effectively allowed to detect GSH through Mn dopant-based QDs (where the ^4^T_1_ → ^6^A_1_ Mn^2+^ ion transition is independent of QDs). It was noticed that the fluorescently dark QDs (OFF state) was observed by capping QDs surfaces with DDTC (an electron rich molecule). Interestingly, the fluorescence was restored (ON state) when the disulfide bond was cleaved by the reducing GSH. They also developed a protocol for the synthesis of water-dispersible ultra-small (3.1 nm) multifunctional CdS:Mn/ZnS core−shell semiconductor QDs and used as fluorescent probes for the bioimaging of TAT-peptide (Gyl-Arg-Lys-Lys-Arg-Arg-Gln-Arg-Arg-Arg-Gly-Tyr-Cys-NH_2_)-conjugated QDs stained in brain parenchyma and blood vessels [31]. In this method, a highly water-dispersible silica layer was formed on each quantum dot (QD) by the hydrolysis and co-condensation reaction of tetraethyl orthosilicate (TEOS), 3-(aminopropyl) triethoxysilane (APTS) and 3-(rihydroxysilyl) propyl methylphosphonate (THPMP). Liu *et al*. applied thioglycolic acid (TGA) capped with CdSe/ZnS QDs as fluorescent probes to detect GSH through OFF–ON principle [32]. They observed OFF fluorescence of QDs by adding methyl viologen; however, the QDs fluorescence was regenerated by the addition of GSH, which is due to a simple ligand displacement process between QDs and target analytes.

### 3.2. Semiconductor QDs as Probes for Analysis of DNA, Bacteria and Cancer Cells

Shih *et al*. used 3-mercaptopropionic acid (MPA) capped with CdS QDs as fluorescent probes for sensing *Salmonella typhimurium* cells [33]. Very recently, Dutta’s team used Mn^2+^-doped ZnS QDs (3–5 nm) as fluorescent probes for detecting *E. coli* [34]. The chitosan has high affinity to bind with the phosphoryl and carboxyl groups of bacterial cell wall. Due to these interactions, chitosan capped QDs can easily penetrate the bacteria cell walls. These results indicate that QDs are potential candidates to measure bacteria cells with high sensitivity. Chang *et al*. [35] synthesized biocompatible chitosan-coated ZnS QDs and chitosan-coated ZnS:Mn^2+^ QDs via a convenient one-step γ-radiation route. These QDs are well dispersed in water and used as sensors for the labeling of PANC-1 cells [35]. Similarly, Shi and Ma described the applicability of TGA capped CdS QDs as fluorescent probes for the detection of DNA molecules (DNA order 5-NH_2_-GAG CGG CGC AAC ATT TCA GGT CGA-3 and a biotinylated complementarity target DNA order of 5-TCG ACC TGA AAT GTT GCG CCG CTC-3) [36]. Since the carboxylic group of TGA (negative charge) has showed high affinity to interact with amidocyanogen probe DNA (positive charge) by the electrostatic interactions. It was noticed that the PL spectrum of CdS QDs-DNA showed a little red shift when compared with TGA capped CdS QDs. The same group described that the utility of fluorescent CdS QDs for the reorganization of DNA molecules through electrostatic interactions [37]. In recent years, visible fluorescent protein constructs provide a significant data to the researchers with a tool of enormous value which can facilitate to the key identification of target molecules in cellular function in tissue sections and in living cells. Clapp’s team described the application of polymer/DNA polyplexes designed water soluble QDs for gene delivery [38]. They found that increasing concentrations of pentablock copolymer and DNA led to quenching of QDs fluorescence, while chloroquine alone had no measurable effect. The designed QDs showed high affinity to monitor the dissociation of pentablock copolymer/DNA polyplexes *in vitro* and were useful for studying the release of DNA within cells.

Recently, intensive research has been carried out on QDs-based sensing probes to develop the tools for the visualization of biomolecules at a single molecule. Because of fascinating light-emitting properties, QDs are offering new exciting opportunities for modern biology to visualize cells *in vitro* and *in vivo*. Wang’s group described an alternative separation-free multiplexed detection method for the detection of low abundance DNA targets, based on multicolor colocalization of target-specific QDs nanoprobes upon hybridization [39]. The designed QDs nanoprobes were successfully used for the genetic analysis of anthrax pathogenicity (*Bacillus anthracis*). Akerman’s group described the applications of ZnS/CdSe QDs with three peptides (GFE-CGFECVRQCPERC; KDE-PQRRSARLSAKPAPPKPEPKP-KKAPAKK; LyP-1-CGNKRTRGC) as nanoprobes to interact with membrane dipeptidase on the endothelial cells in lung blood vessels or blood vessels and tumor cells [40]. These QDs were efficiently recognized lymphatic vessels and tumor cells in cancer related tissues. Similarly, Webb’s group described the use of semiconductor CdSe/ZnSe NCs as fluorescent labels for multiphoton imaging in live animals [41]. These QDs showed high affinity to interact with target analytes for deep *in vivo* multiphoton imaging of living mice. A new class of multifunctional QDs-coated triblock copolymer (polybutyl acrylate, PBA; polyethyl acrylate, PEA; poly(methyl methacrylate), PMMA) was used for linking tumor-targeting ligands and were acted as probes for sensitive imaging of cancer cells *in vivo* conditions [42]. The designed QDs acted as nanoprobes for ultrasensitive and multiplexed imaging of molecular targets *in vivo* and allowed to detect 10–100 cancer cells. Very recently, Wu *et al*. illustrated the use of fluorescent QDs as effective probes for the improved detection and identification of *Staphylococcus aureus* directly from positive blood culture media [43]. These immunofluorescence probes were prepared by capping biotin-conjugated QDs to streptavidin-conjugated IgG molecules. QDs-IgG complex acted as binding agent to interact with a cell surface protein of *S. aureus*. It was found that 73 cultures contain gram-positive *cocci* in clusters and allowed the rapid identification of *S. aureus* directly from specimens. Trioctylphosphine oxide (TOPO) macrocyclic glycocluster amphiphile capped lipophilic CdSe QDs used as water soluble fluorescent sugar ball for probing of Hela cells via endocytosis [44]. The endocytic activity was depended on the sizes of sugar ball QDs, 5 nm size QDs have high endocytic activity more than that of 15 nm and 50 nm sugar ball QDs. Furthermore, Kaul *et al*. [45] described QDs-based fluorescent approach for identification of tumour cells by using labeled QDs with mortalin, and *p*-glycoprotein. These labeled QDs exhibited more photostable than the organic dyes, and allowed to localize *p*-glycoprotein with long fluorescence lifetime.

Recently, engineered QDs used as fluorescent probes for the detection of a wide variety of biomolecules [46,47]. Mattoussi’s group reported the use of CdSe−ZnS core−shell QDs as bioactive fluorescent probes for imaging biomolecules [48]. In this method, lipoic acid was capped on CdSe−ZnS QDs to facilitate electrostatic conjugation of bioactive proteins. This method was successfully used as a powerful fluorescent tracking tool to identify the maltose binding protein in *E. coli*. This method was based on the electrostatic noncovalent interactions between bioconjugates of CdSe/ZnS QDs and antibodies. Goldman’s group described a conjugation strategy for the attachment of antibodies to quantum dots based on the electrostatic interactions between the negatively charged dihydrolipoic acid (DHLA)-capped CdSe-ZnS core-shell QDs and positively charged proteins (natural or engineered) that serve to bridge the quantum dot and antibody [49]. The same group prepared bioinorganic conjugates luminescent semiconductor NCs (CdSe–ZnS core-shell QDs) with antibodies and used as probes in multiplexed fluoroimmunoassays [50]. This system was successfully detected several target species including cholera toxin, ricin, shiga-like toxin 1, and staphylococcal enterotoxin B in single wells of a microtiter plate. Similarly, Chen’s team developed a simple platform for the direct conjugation and separation of highly luminescent CdSe–ZnS QD–antibody complexes using a genetically engineered polyhistidine tagged elastin-protein L fusion [51]. This approach is based on the direct conjugation of QDs via metal coordination with the His tag, and showed high affinity toward IgGs and used as probes for the detection of carcinoembryonic antigen. Huang *et al*. [52] synthesized CdSe/ZnS QDs–ssDNA–fluorescent dye conjugates and used as bioprobes for the detection of micrococcal nuclease activity in the culture medium of *Staphylococcus aureus* by fluorescence microscopy. It has been reported that the QDs are conjugated with ssDNA through streptavidin-biotin affinity ligation. The ssDNA was disrupted in the presence of MNase, which digested the ssDNA.

Many researchers have described the applications of biomolecules (peptides and proteins) capped on QDs for the monitoring alterations of enzymatic activities, which plays key role in biological processes. Luminescent QDs bioconjugates are used to detect proteolytic activity by FRET [53]. Prasuhn’s group functionalized QDs with dye-labeled peptides using two different linkage chemistries to yield FRET for monitoring enzymatic activity or ionic presence [54]. The QDs functionalization is based on carbodiimide chemistry through covalently link dye-labeled peptide substrates to the terminal carboxyl groups on the QD’s surfaces. These QDs showed different chemistries for monitoring chemical and biological molecules. Boeneman *et al*. [55] described the use of a hybrid fluorescent protein semiconductor QDs to monitor caspase 3 proteolytic activity. In this method, mCherry monomeric red fluorescent protein was used to express an *N*-terminal caspase 3 cleavage site and it was ratiometrically self-assembled to the surface of QDs through metal-affinity coordination. The protein capped QD acted as an efficient fluorescence resonance energy transfer acceptor and allowed to monitor the proteolytic activity.

The unique photophysical properties of bioconjugated semiconductor QDs offer many advantages for active sensing, imaging and optical diagnostics [56]. Since, QDs adopted as either donors or acceptors in FRET-based assays and biosensors. Very recently, Algar’s group described the utility of QDs as acceptors and donors within time-gated FRET relays [57]. In this method, the bioconjugated QDs exhibited time-gated energy transfer configuration in prototypical bioassays for monitoring protease activity and nucleic acid hybridization. These QDs also showed dual target format where each orthogonal FRET step transduced a separate binding event. Multiplexed biosensing was performed by exhibiting two approximately independent FRET mechanisms in a single QD-bioconjugate, which is due to spectrotemporal resolution of QDs-FRET without requiring multiple colors of QDs. Morgner’s group reported a method on time-resolved multicolor optical analysis of FRET from a luminescent terbium complex to different semiconductor QDs [58]. This method is an efficient platform for rapid and accurate measurement on size and shape of various quantum dots under physiological conditions. It provides a new platform for the simultaneous analysis of different biological processes at sub-nanomolar concentrations. The same group described FRET mechanism between luminescent terbium complexes (LTC, as donors) to semiconductor QDs (as acceptors) for creating extraordinary large FRET efficiencies [59]. This system permits an efficient suppression of autofluorescent background which facilitates to detect analyte at sub-picomolar. Their unique physico-chemical properties make them as excellent candidates for the sensing biomolecules. The luminescent terbium complexes-QD FRET assays confirmed that conjugated QDs have well ability to specific interaction with biotin-streptavidin or the metal-affinity coordination of histidine to zinc. Similarly, luminescent terbium complexes containing different semiconductor QDs used as probes for the multiplexed bioassay with sub-picomolar detection limits for bioanalytes [60]. Using this probe, the sensitivity of the method was greatly increased and allowed to detect single-analyte assay.

Due to their unique optical properties, semiconductors QDs have been used as central photoluminescent scaffolds for a wide variety of biosensing platforms. For example, the aptamer-capped near-infrared PbS QDs were used as a probe to detect a target protein based on selective charge transfer [61]. In this method, the water-soluble QDs were functionalized with thrombin-binding aptamer, which retains the secondary quadruplex structure for binding to thrombin. The functionalized PbS QDs were effectively interacted with thrombin with detection limit of 1 nM. This fluorescence quenching mechanism was based on charge transfer from the amine groups of protein to QDs. Similarly, quantum-dot aptamer beacons were used as fluorescence probes for sensitive detection of protein targets, such as thrombin and nucleic acid targets [62]. Rao and co-workers described the utility of CdSe/ZnS core-shell structures to identify protein-ligand interactions through BRET [63]. It was noticed that QDs were reacted with 1000 equivalents of HaloTag ligand, and then reacted with increasing concentrations of HTP–Luc8. As a result, the QDs-conjugates showed increasing bioluminescence emissions both from HTP–Luc8 and from the QDs. This approach is based on BRET where QDs act as energy acceptors for the light emitting protein (e.g., bioluminescent protein *Renilla luciferase*). The same group developed semiconductor QDs-based bioluminescence resonance energy transfer (QDs-BRET) approach for the sensing of protease activity in complex biological samples [64]. In this method, nanosensors consist of bioluminescent proteins as the BRET donor, quantum dots as the BRET acceptor and protease substrates sandwiched between the two as a sensing group. This system was successfully used to detect matrix metalloproteinases. Ma *et al*. synthesized multicolor quantum QD-encoded microspheres via layer-by-layer (LbL) assembly approach and polyelectrolyte multilayers are assembled on the QDs surfaces via electrostatic interactions [65]. In this method, two kinds of biofuntional multicolor microspheres are prepared by using two different antibodies (anti-human IgG and anti-rabbit IgG). This approach (human IgG and rabbit IgG) showed high ability to detect target antigens by the multiplexed fluoroimmunoassays.

Functionalized QDs have been widely used as fluorescent probes in the fields of biomedical and biosensing research owing to their stable and tunable multicolor fluorescence, broad absorption with narrow emission spectra, large molar extinction, high quantum yield and high chemical stability. For example, folate (FA) was attached onto the water-dispersible amphiphilic polyethylene glycol (PEG)-coated QDs (FA-PEG-QDs) via hydrophobic interactions [66]. These QDs used as fluorescent nanobioprobes for the reorganization of folate receptors (FRs) were over expressed in human nasopharyngeal cells (KB cells) but not in an FR-deficient lung carcinoma cell line (A549 cells). Similarly, Liu’s group illustrated the carbodiimide coupling chemistry to prepare QDs-hydrophobic ligands through covalent modification [67]. The QDs were functionalized with a new class of hetero bifunctional ligands by incorporating different molecules such as dihydrolipoic acid, a short poly(ethylene glycol) (PEG) spacer and an amine or carboxylate terminus and subsequently attached a RhB dye to form QDs-dye conjugate FRET. These engineered QDs showed high-affinity towards target cells through covalent attachment, which facilitates to track biotinylated epidermal growth factor (EGF) in live cells. Seeberger’s group described the utility of PEGylated QDs capped with carbohydrates (D-mannose, D-galactose, and D-galactosamine) as fluorescence probes to investigate specific carbohydrate−protein interactions *in vitro* and *in vivo* in liver [68].

**Figure 3:**
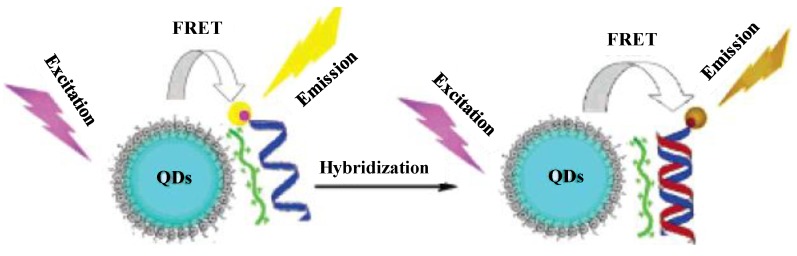
Mechanism of DNA hybridization-detection System based on the QDs/Cy3-labeled DNA Förster (Fluorescence) Resonance Energy Transfer (FRET). Reprinted with permission from [70].

Recently, QDs have been functionalized with various ligands and used as fluorescent probes to detect a wide variety of biomolecules. For example, Zhang *et al*. [69] developed an ultrasensitive nanosensor platform to detect low concentrations of DNA in a separation-free format. The mechanism was based on fluorescence resonance energy transfer between QDs and the linked DNA. In this approach, QDs acts as concentrator to amplify the target signal by confining several targets in a nanoscale domain. This method was successfully used to detect a point mutation typical of some ovarian tumors in clinical samples. Similarly, Peng’s group described the potential application of poly(diallyldimethylammonium chloride) (PDADMAC) capped CdTe QDs as probe for DNA-sensing [70]. This approach is based on FRET between blue-luminescent CdTe QDs and dye-labeled ssDNA (Figure 3). In this method, a cationic polymer was used as an “electrostatic linker” to achieve efficient energy transfer from the QD donor to the dye acceptor. Similarly, dodecanoic acid-(DDA)-, hexadecylamine (HAD)- and tri-*n*-octylphosphine oxide-(TOPO) capped CdSe/ZnS QDs [71] and streptavidin functionalized CdSe/ZnS and iron oxide NPs [72] have been used as fluorescent probes to identify DNA. These QDs-based fluorescence approaches allowed to detect ultra-trace target DNA within few minutes and offered the detection limit of 1.0 × 10^−9^ M. This platform provides a homogeneous DNA assay for fluorescence detection of DNA with minimal modification. Biomolecules were successfully functionalized on the QDs to detect bacteria. Briefly, TGA [73] and bovine serum albumin (BSA) [74] capped CdSe QDs were used as fluorescent probes for rapid detecting *Escherichia coli* and *Staphylococcus aureus*. The TGA functionalized CdSe QDs showed high degree of interaction with the amino group of bacterial cell membranes of *E. coli* and *S. aureus* and detected the bacteria at 10^2^–10^7^ CFU/mL. Similarly, BSA-conjugated CdSe QDs provided new strategy for the cell labeling with good biologically-compatible materials and this strategy was based on the interaction between BSA–CdSe or CdSe/CdS QDs and cells. The BSA-coated QDs strongly influenced the physical properties of membranes and decreased the cell membrane fluidity and the cell surface of *E. coli*. Singh’s group applied TGA-capped CdSe/ZnS core/shell QDs to detect *Salmonella typhi* [75] in which the carbodiimide chemistry was utilized to conjugate *S. typhi* through carboxy terminated TGA capped QDs. This mechanism was based on the sandwich fluoroimmuno sensing between antibody immobilized membrane and bioconjugated QDs.

### 3.3. Semiconductor QDs as Probes for Analysis of Peptides and Proteins

**Figure 4:**
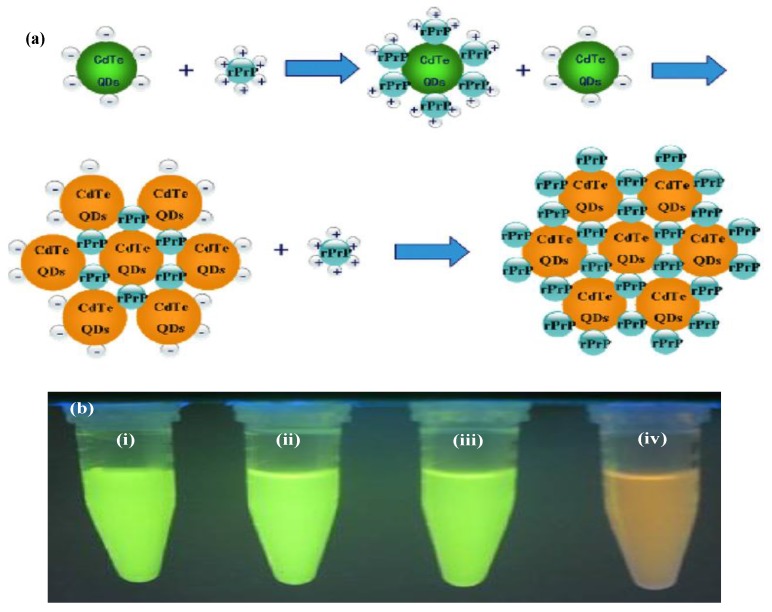
(**a**) Interactions of CdTe QDs with rPrP; (**b**) Colorimetric qualitative detection of rPrP (6.7 µg mL^−1^) with CdTe QDs (1.0 × 10^−6^ mol L^−1^). The excitation wavelength of UV lamp was 302 nm. (i) CdTe QDs; (ii) CdTe QDs with BSA; (iii) CdTe QDs with His6-tagged MBP and (iv) CdTe QDs with rPrP. Reprinted with permission from [76].

To efficiently enhance the specificity and sensitivity of fluorescence signal, it is necessary to functionalize QDs with organic or biomolecules to act as recognition and signal triggering elements for biomolecules assays. For example, Zheng’s group prepared mercaptoacetic acid (MAA) capped CdTe QDs and used as fluorescent probes for the detection of prion protein (rPrP) [76]. These QDs showed high selectivity towards rPrP and the emission wavelengths were observed at 551 nm for original CdTe QDs and at 558 nm for CdTe QDs-conjugated rPrP, respectively. This is due to the shorter distances between the CdTe QDs in the protein conjugates than the free CdTe QDs, which can increase the dipole-dipole interaction between the CdTe QDs resulting in a larger Stoke’s shift emission change (Figure 4a). The bare QDs solution emitted bright green fluorescence; however, bright green fluorescence was completely changed to orange emission by the addition of rPrP into CdTe QDs solution, which can observe with naked eye (Figure 4b). The authors studied the fluorescence abilities of CdTe QDs with various biomolecules (rPrP, BSA and His6-tagged maltose binding protein). It was found that only CdTe QDs–rPrP solution exhibited orange emission (Figure 4b).

The applications of QDs are vastly diverse with tremendous outcome in multidisciplinary research. Many QDs have been functionalized with various capping agents to sensor proteins. For instance, citrate- [77] and diethanolamine- [78] capped CdSe QDs were used as fluorescent probes to detect cytochrome c, hemoglobin, myoglobin and BSA. The CdSe and CdSe/CdS have well-defined 1s–1s electron transitions and the fluorescence intensity of cytochrome c (2 × 10^−9^ M) was significantly quenched by the exposure of CdSe/CdS core-shell QDs. This quenching mechanism was based on the photo-induced electron transfer between QDs and cytochrome c because it is an electron transfer protein. Similarly, the flower-like CdSe nanostructure particles were used as fluorescent probes to detect BSA [78]. CdSe QDs fluorescence intensity was strongly quenched by the interaction of CdSe QDs with tryptophan residues. The BSA structure was greatly changed by interaction with CdSe QDs. It is a static quenching phenomenon between the quencher (CdSe QDs) and the fluorophore (BSA). CdSe/ZnS core-shell QDs were used as optical sensors to investigate optical and structural properties of QDs conjugated with BSA [79]. Curri’s group modified the procedure for preparation of CdSe/ZnS core-shell with hydrophobic ligands (DSPE-PEG-2000—1,2-distearoyl-sn-glycero-3-phosphoethanolamine-N-[carboxy(polyethylene glycol)-2000]) [79]. The functionalized CdSe/ZnS QDs showed strong tendency to interact with BSA through amide bond between functionalized QDs/PEG lipid micelles and the primary amine groups of the BSA. These BSA conjugated QDs can be used as optical probes for multi-color and multiplexing bio-probes detection and also can be used for molecular recognition processes. Furthermore, the TGA-coated CdTe QDs were used as fluorescent probes to study the optical properties of BSA [80]. This conjugation is based on the electrostatic interactions between the carboxylic group of TGA with the amino group of BSA. It was noticed that the fluorescence intensity of QDs was gradually increased with the increasing amount of BSA. To quench the fluorescence intensity, three surfactants were added into the QDs-BSA conjugated system. The maximum quenching efficiency was observed in the presence of cetyltrimethylammonium bromide (CTMAB). This method demonstrated the high performance QDs-NR FRET sensor for ultra sensitive detection of target species with low background signals. To increase the fluorescence quenching, He’s team prepared TGA- coated CdTe QDs (2–3 nm) and used as optical probes to study the interactions between CdTe QDs and chymotrypsin by UV-visible, fluorescence and resonance Rayleigh scattering tools [81]. It was obvious that CdTe QDs successfully quenched the intensity of chymotrypsin at pH 7.2 via static quenching. The same group described the application of 2-mercaptoethylamine (MEA) capped CdTe QDs as fluorescent probes to investigate QDs interactions with human serum albumin (HAS) [82]. The fluorescence intensity of HSA was strongly quenched by the addition of CdTe QDs due to the electrostatic interactions between the positive charge of MEA-CdTe QDs and the negative charge of HSA.

Very recently, Jiang’s and Liu’s teams synthesized CdTe QDs with different ligands such as mercaptopropionic acid (MPA), N-acetylcysteine (NAC) and GSH used as optical probes to investigate the HSA structures by using fluorescence spectroscopy, circular dichroism (CD), UV–vis spectroscopy and dynamic light scattering [83]. In this method, both the static and dynamic quenching mechanisms occurred through electrostatic interactions between QDs and HSA. Wei’s group investigated the influence of CdTe QDs size on the toxic interaction with HSA [84]. In this method, two aqueous-compatible CdTe QDs with maximum emission of 535 nm (green-emitting QDs, G-QDs, 2.04 nm) and 654 nm (red-emitting QDs, R-QDs, 3.79 nm) were used and it was concluded that the quenching effect of QDs on HSA fluorescence depended on the size. They confirmed that the nature of quenching is not dynamic but probably static, resulting in forming QDs–HSA complexes. Similarly, Huang’s and Liu’s groups prepared MPA capped CdTe QDs and used as fluorescent probes for the investigation of optical properties of HSA using fluorescence spectroscopy [85]. In this method, four sizes of MPA capped CdTe QDs with maximum emission of 520 nm (green QDs), 568 nm (yellow QDs), 620 nm (red QDs) and 680 nm (crimson QDs) were studied and found that the quenching of HSA fluorescence intensity is QDs size-dependent relationship. This quenching is based on the electrostatic interaction between QDs and HSA, which facilitate to form QDs–HSA complex. It was noticed that the biological activity of HSA was very poor at bigger sizes of QDs. Wang’s and Liu’s teams studied the interaction of CdTe QDs coated with MPA, L-cysteine, and GSH with BSA by fluorescence, UV–vis absorption and circular dichroism [86]. The functionalized CdTe QDs effectively quenched the fluorescence intensity of BSA and the interactions were based on the electrostatic interactions, which results to form QDs-BSA complexes. These results play a key role to understand QDs-proteins interactions in proteomics.

Recently, several fluorescent semiconductor QDs have been synthesized and applied as fluorescent probes in proteomic studies. Briefly, L-cysteine-capped ZnS [87] and MAA-capped ZnSe [88] QDs were used as fluorescent probes for analysis of several proteins by fluorescence spectroscopy. The fluorescence intensity of L-cysteine capped ZnS QDs was increased by the addition of proteins such as BSA, HSA, γ-globulin, ovalbumin at 267 nm in pH 5.12 [87]. Similarly, the fluorescence intensity of MAA capped ZnSe QDs was greatly enhanced by the addition of BSA at pH 7.0 and the limit of detection was 0.06 nM [88]. The fluorescence intensity of QDs was enhanced by the presence of BSA and emitted a characteristic peak at 348 nm. As a result, strong interactions occurred between the functionalized ZnSe with BSA. Furthermore, *p*-sulfonatocalix [4,6] arene could be used as a stable ligand to prepared water-soluble nanoparticles via chemical bonding. As a result, Li and co-workers described a simple procedure for the preparation of highly fluorescent, stable and water-soluble CdSe QDs functionalized with *p*-sulfonatocalix(n)arene (SFCA, n = 4 or 6) and used as fluorescent probes for the detection of aminoacids [89]. It was found that the SFCA(4) coated CdSe QDs showed high selectivity towards methionine, similarly, SFCA(6) coated CdSe QDs showed high affinity towards phenylalanine. The fluorescence signal intensity of SFCA(n) coated with QDs was greatly enhanced in the presence of methionine and phenylalanine. Based on the above reports, we assert that the surface of QDs needs to be altered to the increase stability and allow conjugation of biomolecules for specific targeting. Table 1 indicates the semiconductor QDs-based fluorescence spectrometric methods for the analysis of biomolecules.

**Table 1:** Detection of biomolecules using semiconductor QDs-based fluorescence spectroscopic methods.

Name of QDs	Name of the ligand	Diameter (nm)	Analytes	Detection limit (nM)	Reference
CdS:Mn/ZnS	DDTC	5	GSH	-	[30]
CdS:Mn/ZnS	Amine group	3.1	TAT-peptide	-	[31]
CdSe/ZnS	TGA	-	GSH	-	[32]
CdS	MPA	2–5	*Salmonella typhimurium* cells	-	[33]
Mn^2+^ZnS	Chitosan	3–5	*E. coli*	-	[34]
Mn^2+^ZnS and ZnS	Chitosan	4.5	PANC-1 cell	-	[35]
CdS	TGA	5	DNA	-	[36]
CdSe/ZnS	Peptides (GFE, KDE, LyP-1)	<10	Tumors	-	[40]
CdSe/ZnS	PBA, PEA, PMMA	2.5–5.0	Cancer cell	10–100	[42]
CdSe	TOPO-sugars	5–50	Hela cell	-	[44]
CdSe/ZnS	FA-PEA-PEG-750	13	KB *vs*. A549 cells	-	[66]
CdSe/ZnS	Amino, carobxy and hydroxy groups PEG	-	Hela cell	-	[67]
CdSe/ZnS	PEG- D-mannose, D-galactose, and D-galactosamine	15–20	Hepatocellular carcinoma cell line HepG2	-	[68]
QDs	Streptavidin	-	DNA	4.8 × 10^−15^	[69]
CdTe	PDADMAC	-	DNA	-	[70]
CdSe/ZnS	TOPO-DDA, TOPO-HDA	3.3	DNA	<1.0 × 10^−9^	[71]
CdSe/ZnS and iron oxide NPs	Streptavidin	30	DNA	5.0 × 10^−6^	[72]
CdSe	TGA	3–4	*E. coli* and *S. aureus*	10^2^ CFU/mL	[73]
CdSe	BSA	5	*E. coli* (HB101)	-	[74]
CdSe/ZnS	TGA-IgG	6	*Salmonella typhi*	10^2^ cells/mL	[75]
CdTe	MAA	~3.4	rPrP, *E. Coli*	3.0	[76]
CdSe and CdSe/CdS	Citrate	-	cytochrome c, hemoglobin and myoglobin	-	[77]
CdSe	Diethanolamine	20	BSA	-	[78]
CdSe/ZnS	DSPE-PEG-2000	3.5–4	BSA	-	[79]
CdTe	TGA-neutral red-BSA	3.1	BSA	1.97	[80]
CdTe	TGA	2–3	Chymotrypsin	-	[81]
CdTe	MEA	-	HSA	4.2	[82]
CdTe	MPA, NAC, GSH	-	HSA	-	[83]
CdTe	MPA	2–4.8	HSA	-	[85]
CdTe	MPA, L-Cys, GSH	3.5	BSA	-	[86]
ZnS	L-Cys	17	BSA, HSA, γ-globulin, ovalbumin	0.06–0.56	[87]
ZnSe	MAA	25	BSA	30.3	[88]
CdSe	SFCA ( *n* = 4, 6)	-	Methionine and phenylalanine	3000–4000	[89]

## 4. Semiconductor Nanomaterials-Based MALDI-MS for Biomolecules Analysis

**Figure 5:**
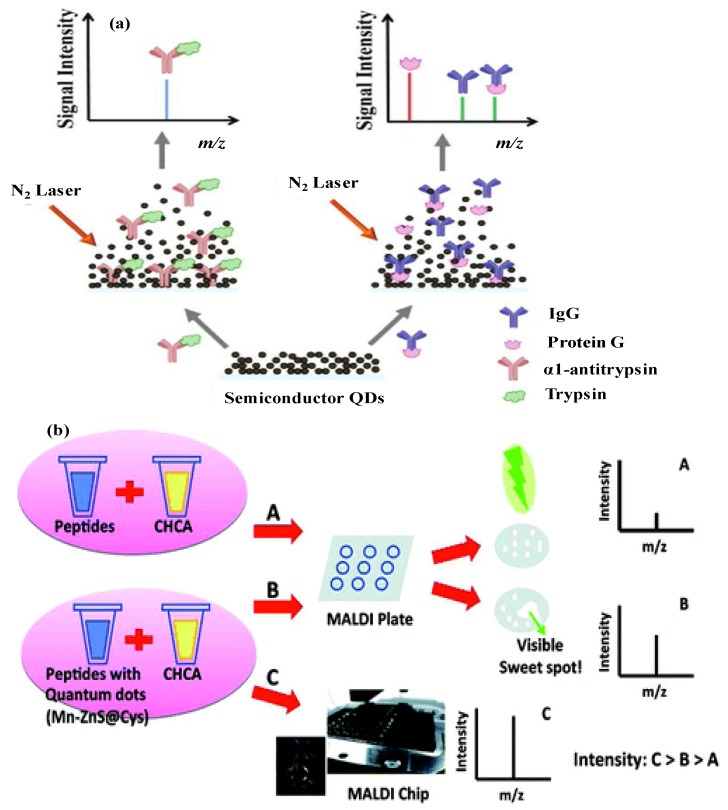
(**a**) Schematic representation of QDs as matrices for analysis of biocomplex structures by laser desorption ionization (LDI)-MS. Reprinted with permission from [90]; (**b**) Schematic diagram of peptide analysis by using hydroxycinnamic acid (CHCA), QDs along with CHCA and QDs based matrix-assisted laser desorption/ionization (MALDI) chip along with CHCA. Reprinted with permission from [91].

In MALDI-MS, the organic matrices such as 2,5-dihydroxybenzoic acid (DHB), α-cyano-4-hydroxycinnamic acid (CHCA) and sinapinic acid (SA) act as energy mediators for laser absorption and transferring of laser energy to target analytes for their desorption and ionization, which minimize the analytes damage and fragmentation from laser irradiation. Generally, a good matrix exhibits good ability to form co-crystallization with target analytes, which leads to generate a layer of small crystals. By applying nitrogen (N_2_) laser at 337 nm, the analytes get evaporate and leads to create gas phase ions and then can be identified by mass spectrometry (Figure 5a). Although organic matrices acted as potential candidates for analyzing large biomolecules (peptides and proteins) by MALDI-MS, there are some problems associated with the use of organic matrixes such as strong interference at low mass region and inability to enrich trace level target molecules. Since the organic matrices typically form heterogeneous crystallization to create ‘‘sweet spots’’, resulting in poor shot-to-shot and sample-to-sample reproducibility. Recently, various types of nanomaterials have been applied in MALDI-MS for the analysis of a wide variety of molecules. Since, nanomaterials have large surface areas for large analyte loading capacity (e.g., >1000 small molecules per single nanoparitcle), and surface chemistry of nanomaterials allows us to act as affinity or concentrating probes for the enrichment or preconcentration of target molecules in biocomplex samples (Figure 5b). Because the number of adsorbed analytes can be increased with decreasing size of nanomaterials, greater desorption/ionization efficiency with higher sensitivity is provided.

Tanaka’s group first demonstrated the potential use of cobalt powder (30 nm) dispersed in glycerol as the inorganic matrix for the analysis of proteins by MALDI-MS [92]. Nanomaterials surfaces exhibit several unique properties such as higher analyte loading capacities, high molar absorption coefficients, efficient absorption of laser light, minimal degree of fragmentation with reduced background noise and high affinity to enrich target species from sample solutions. In recent years, scientific interest towards the chemistry of semiconductor nanomaterials have been rapidly grown in multidisciplinary research areas and successfully integrated with mass spectrometric tools for the analysis of biomolecules [93]. Since nanomaterials integrated MALDI-MS possesses distinctive advantages such as sensitivity, enrichment of trace amounts of targets and minimization of sample pretreatment procedures to the biomolecules assays [94,95]. In response to these advantages, a variety of nanomaterials including metal and metal oxide nanoparticles, semiconductor nanoparticles and QDs have been introduced as matrices and affinity probes for the efficient analysis of biomolecules by MALDI-MS. However, this review provides a general overview of recent approaches on semiconductor nanomaterials-based MALDI-MS for the analysis of biomolecules.

### 4.1. Metal Sulphide Semiconductor NMs-Based MALDI-MS

**Figure 6:**
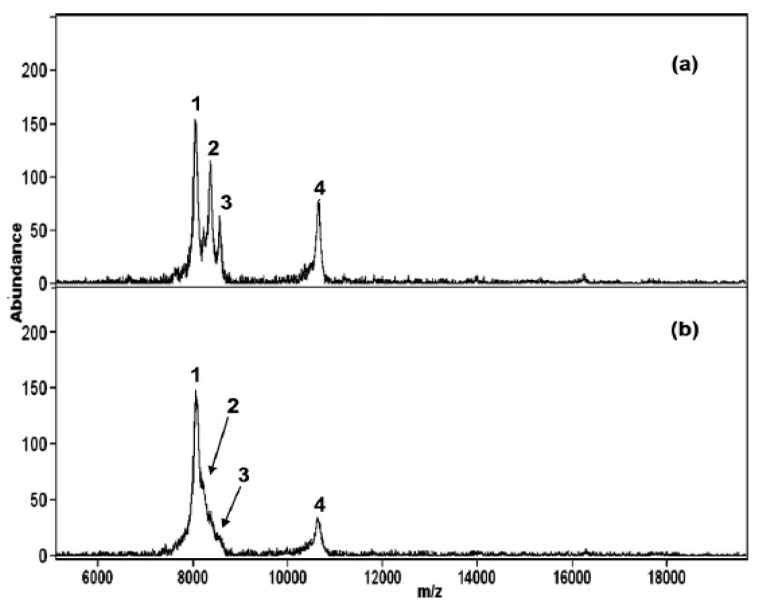
MALDI-mass spectra of ubiquitin-like proteins in oyster mushroom by (**a**) ZnS-MPA NPs as the matrix and affinity probes; and (**b**) sinapic acid (SA) as the matrix. Peaks 1, 2, 3, and 4 at *m/z* 8063, 8384, 8593, and 10 666 are attributed to the ubiquitin-like protein (8.0 kDa), ubiquitin-like protein (8.3 kDa), ubiquitin (8.5 kDa), and ubiquitinlike protein (10.5 kDa), respectively. Reprinted with permission from [96].

Due to their unique physico-chemical properties, semiconductors-based nanomaterials have been intensively used in MALDI and matrix-less laser desorption ionization (LDI) mass spectrometry; the ultimate objective is to overcome several serious limitations (reproducibility, enrichment of trace level analytes, and elimination of background noise) intrinsically related to the use of conventional organic matrices in MALDI-MS [93,94,95]. Therefore, Wu’s group introduced various functionalized semiconductor nanomaterials and used as matrices, affinity probes and heat absorbing probes for the analysis of peptides, proteins and bacteria by LDI- and MALDI-MS [96,97,98,99,100,101,102,103,104,105]. Briefly, the ZnS NPs were functionalized with five different functional groups (MPA, sodium citrate, cysteamine, and 2-mercaptoethane sulfonate) and used as affinity probes for the analysis of proteins by MALDI-MS [96]. It was noticed that MPA functionalized ZnS NPs were effectively acted as affinity probes to concentrate ubiquitin-like proteins from oyster mushroom (*Pleurotus ostreatus*). In this approach, MPA capped ZnS NPs have showed high affinity towards ubiquitin-like proteins (Figure 6). Similarly, azide capped ZnS-N_3_ NPs used as separating and affinity probes (separation-/washing free) to isolate/concentrate milk proteins and ubiquitin like proteins from oyster mushroom prior to their identification by MALDI-MS [97]. The separation and preconcentration phenomenon was based on the electrostatic interaction between ZnS-N_3_ NPs surfaces and proteins. Because of their inherent advantages, Mn^2+^-doped ZnS NPs functionalized with cysteine and used as matrix for the analysis of small molecules [98]. Since, Mn^2+^-doped ZnS NPs have potentiality to absorb a greater number of molecules on their surfaces over those of traditional organic matrixes, which facilitates to generate more number of analytes desorption/ionization from NPs surfaces per laser shot, offering a greater sensitivity.

**Figure 7:**
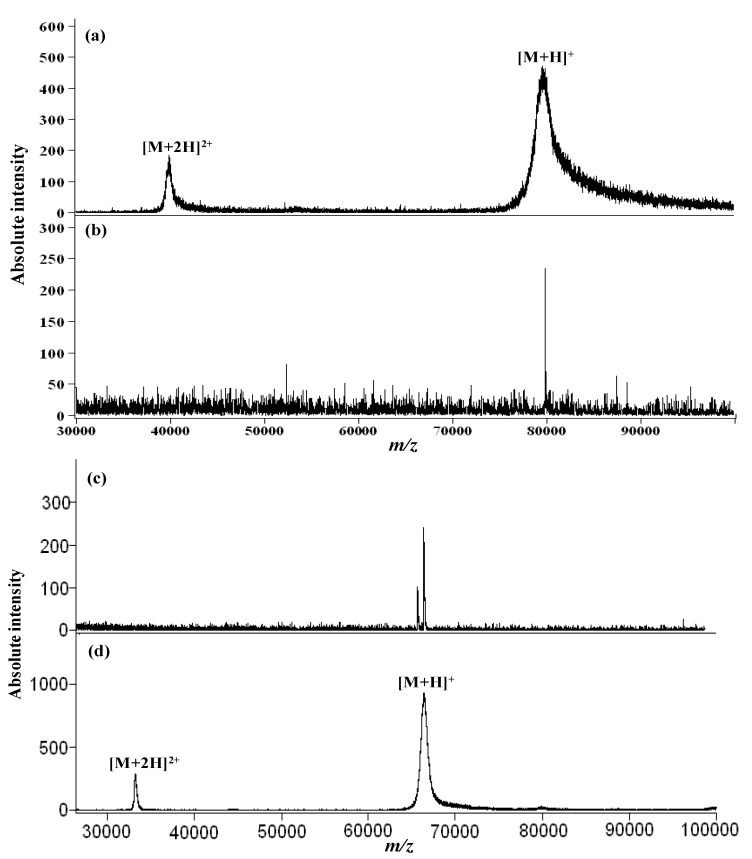
MALDI—mass spectra of transferrin using (**a**) SA; and (**b**) CdS QDs as the matrices. MALDI—mass spectra of bovine serum albumin (BSA) using (**c**) CdS QDs; and (**d**) SA as the matrices. Reprinted with permission from [99].

Semiconductor CdS QDs and NPs functionalized with MPA [99,100] and 4-aminothiophenol (ATP) and 11-mercaptoundecanoic acid (MUA) [100] and used as matrices and affinity probes for the analysis of biomolecules by MALDI- and ESI-MS. The MPA capped CdS QDs were covalently interacted with large proteins and the mass resolution of large proteins (bovine serum albumin, human serum albumin and transferrin, 66,000–80,000 Da) was greatly enhanced by acting as efficient energy mediators (Figure 7). Furthermore, MPA capped CdS QDs used as affinity probes for the enrichment of microwave digested proteins (cytochrome c and lysozyme) prior to their identification by ESI-MS [100]. Semiconductor CdS NPs capped with ATP and MUA used as matrices and as co-matrices for the analysis of peptides and proteins in MALDI-MS [101]. It was observed that the -NH_2_ group of ATP and COOH group of MUA on CdS NPs acted as a supporting matrix for the efficient transfer of protons to target analytes during their desorption/ionization. Using these materials, peptides and proteins signals were generated with high signal intensities with reduced background noise and increased mass resolution (4–13 folds). These ATP- and MUA- capped CdS NPs used as affinity probes to isolate/concentrate hydrophobic proteins (soybean hydrophobic protein, soybean Bowman–Birk proteinase inhibitor and soybean inhibitor D-II) in soybean prior to their identification by MALDI-MS. The potential of MUA capped CdSe QDs-assisted LDI-MS as a new tool for proteomic studies [102]. The functionalized CdSe QDs acted as an effective probe to interact with biomolecules through covalent interactions. This method greatly enhanced the signal intensities of proteins and the LOD of peptides was found to be 100 pM. Importantly, the MUA capped CdSe acted as concentrating probes in the presence of signal suppressors such as urea and Trition X-100. Multiwalled carbon nanotubes doped with Cd^2+^ ions and modified with CdS NPs used as affinity probes for the analysis of peptides and microwave-digested proteins in the atmospheric pressure matrix-assisted laser desorption/ionization (AP-MALDI) and MALDI MS [103]. Moreover, these NPs also acted as heat-absorbing materials for efficient microwave tryptic digestion of cytochrome c and lysozyme in MALDI-MS.

### 4.2. Metal Selenide, Telluride and Oxide Semiconductor NMs-Based MALDI-MS

**Figure 8:**
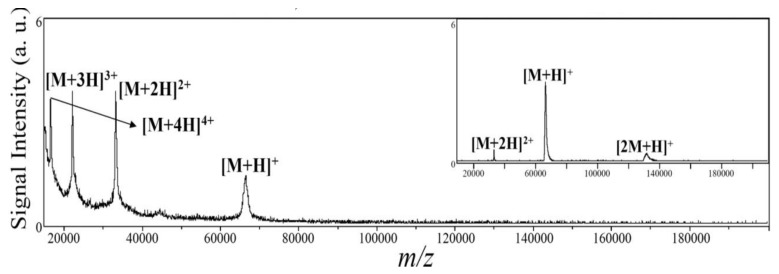
Mass spectrum of BSA (5 µM) recorded using a matrix of HgTe nanostructures prepared in 300 mM ammonium citrate (pH 5.0). The signals at *m/z* 66 431, 33 216, 22 144, and 16 609 represent the adducts [M + H]^+^, [M + 2H]^2+^, [M + 3H]^3+^, and [M + 4H]^4+^, respectively. Inset: Mass spectrum of BSA obtained in SA matrixes (20 mg/mL in water/ACN (1:1, *v/v*) containing 0.1% TFA). The signals at *m/z* 132 862, 66 431, and 33 216 represent the adducts []^+^, [M + H]^+^, and [M + 2H]^2+^, respectively. Reprinted with permission from [90].

Furthermore, MPA capped ZnSe QDs were used as the matrix and affinity probes for direct analysis of peptides and proteins from sodium salt solution in SALDI-MS [104,105]. The signal enhancement was owing to the electrostatic interaction between QDs and biomolecules, which facilitate the adsorbtion proteins onto the surfaces of ZnSe QDs. Using this method, several biomolecules such as Leu-enk, Met-enk, HW6, substance P and angiotensin II, cytochrome c, myoglobin and lysozyme were successfully analyzed by MALDI-MS. Moreover, these approaches provided straightforward tool for the identification of peptides, proteins and digested proteins with reduced sample preparation procedures. It is well known that the organic matrices can create invisible “sweet spots” that are formed during heterogeneous cocrystallization which can minimize the analytical throughput and affect the reproducibility of MALDI-MS. To solve this problem, Lai’s group described the potential applications of cysteine capped Mn^2+^–ZnS QDs-assisted MALDI-MS for peptide and protein analysis [91]. These functionalized QDs NPs acted as affinity probes for selective trapping of target biomolecules through strong interactions between analytes and the cysteine capping agents on the surfaces of QDs. Due to their small size and high surface-to-volume ratio, biomolecules are strongly adsorbed on the surfaces of QDs, which can increase relative peptides concentration. Importantly, Mn^2+^-ZnS QDs acted as nucleation centers for the formation of visible sweet spots during matrix crystallization and permitted to enhance the signal intensities of target molecules at 1.0 pM. Similarly, Chang *et al*. [90,106] reported the tremendous applications of HgTe nanostructure materials as matrices and affinity probes for the analyses of large proteins complexes (150,000 Da) (Figure 8). In these methods, several influencing parameters such as nanostructure, surfactant, pH and salt concentrations were investigated for the analysis of large proteins by SALDI-MS with high sensitivity. It was observed that BSA protein signals were generated by using ammonium citrate (pH 5.0). Due to their weaker interaction (BSA (pI 4.5) and HgTe nanostructures (zeta potential, −37.6 mV)), efficient desorption occurred for the generation of large proteins signals. Apart from this, HgTe nanostructure acted as concentrating probes, which facilitated to decrease LODs by a factor of all most 50 times [90]. Similarly, they also described the use of MPA capped HgTe nanostructures as the matrix for the analysis of large proteins (α1-antitrypsin, trypsin, IgG, protein G) and their complexes by SALDI-MS [106]. They studied the role of several surfactants (PEG 300, PEG 600, PEG 2000, Tween 20, Brij 30, Brij 35, Brij 56, and Brij 76, 1%) for the detection of protein-protein complexes by SALDI MS. It was noticed that only Brij 76 was provided the best conditions for the detection of protein-protein complex ions [α_1_-antitrypsin+trypsin+H]^+^ at *m/z* 72,160. This is due to Brij 76 surfactant has high hydrophobicities (nonpolar alkyl chains and OH groups), which allow to stabilize the protein-protein complexes. Using this method, the interactions of IgG and protein G proteins were investigated and the signals were observed at *m/z* 74,885, 49,984, 26,023, 13,019, 86,585, and 58,297 corresponding to [IgG + 2H]^2+^, [IgG + 3H]^3+^, [protein G + H]^+^, [protein G + 2H]^2+^, [IgG + protein G + 2H]^2+^, and [IgG + protein G + 3H]^3+^ adducts, respectively. This HgTe-based SALDI-MS allowed to detect protein G (*m/z* 26,023) and IgG (*m/z* 149,931) at 2 µM (1 pM) and 5 µM (2.5 pM) with ease sample preparation at minimal sample volume. Very recently, Kailasa and Wu described that the potential applications of DDTC capped QDs [107] and the bare BaTiO_3_ NPs [108] as affinity probes for the enrichment of microwave tryptic digest proteins (cytochrome c, lysozyme and BSA and phosphoproteins) prior to MALDI-MS analysis. Using QDs as affinity probes, proteolytic peptides of aminoacid sequence coverages were observed with 92%, 75% and 56% for cytochrome c, lysozyme and BSA, respectively [107]. Similarly, BaTiO_3_ NPs-assisted MALDI-MS provides higher digestion efficiency and enriched maximum number of phosphopeptides from the tryptic digest phosphoproteins [108]. Overall, these approaches provided rapid tools for digestion of proteins (50 s) and effectively trapped/enriched proteolytic peptides in 30 min, which is promising for automated high-throughput proteomics studies in modern biology.

Xiong’s group described a new method for the synthesis of SnO_2_- poly(methyl methacrylate) (PMMA) and TiO_2_-PMMA core–shell nanobeads and used as affinity probes to concentrate intact horse heart myoglobin (400 nm) and trypsin-digested myoglobin peptides (1 nm) prior to SALDI-MS [109]. The SnO_2_-PMMA nanobeads showed the superior qualities to concentrate and enrich target analytes in SALDI MS than that of TiO_2_-PMMA. Since, SnO_2_ NPs (2 nm) have smaller size than TiO_2_ NPs (8 nm), which permits higher capacity to trap/concentrate proteins from the brain of Sprague–Dawley rat. Chang’s [110] team described the utility of six nanomaterials (Au, TiO_2_, Se, CdTe QDs, Fe_3_O_4_, and Pt nanosponges) as matrices for the analysis of glutathione, angiotensin I, insulin, cytochrome c and chymotrypsin by SALDI-MS. Large protein (cytochrome c) signals were generated with a greater number of adducts using Fe_3_O_4_ NPs and CdTe QDs as SALDI substrates because Fe ions desorbed and ionized from the NPs than those from the mixtures of the metal ions. The limit of detection was 5.6 fM for cytochrome c using Fe_3_O_4_ NPs as the matrix. Based on these results, we assert that NPs need to meet the three criteria such as (i) high ability to absorb the laser energy; (ii) prevent analyte aggregation and well co-crystallization; and (iii) provide a source of charging (e.g., H^+^). Therefore, these semiconductor nanomaterials-based MALDI-MS approaches offer a powerful bioanalytical tool for ultra-fast analysis of trace level proteins.

### 4.3. Semiconductor Nanomaterials-Based MALDI-MS for Bacteria Analysis

To enhance the specificity and sensitivity of the bacteria proteins signals, it is necessary to seek suitable methods for the functionalization of NPs with organic or biomolecules as affinity probes and signal triggering elements. To improve the signal intensities of bacteria proteins, our group published a series of papers on NPs-assisted MALDI-MS for the analysis of bacterial proteins in various bacteria species [111,112,113,114,115,116]. Several nanomaterials such as Mg(OH)_2_ [111], ZnO [112,113], CdS QDs [114], TiO_2_ [115], and titanium-based biochip [116] have been used as affinity probes/concentrating probes for the enrichment of bacteria proteins prior to MALDI-MS analyses. In these approaches, oleic acid capped Mg(OH)_2_ NPs were used as extracting and concentrating probes for the enrichment of hydrophobic proteins in *E. coli* before their identification by MALDI-MS [111]. Similarly, ZnO NPs [112] acted as affinity probes for the analysis of bacteria proteins in *E. coli* [112] and in *Staphylococcus aureus* [113]. The degradative ability of CdS QDs was investigated on extracellular polysaccharides of *E. coli* cells [114]. Similarly, TiO_2_ NPs-assisted MALDI-MS used as a biosensor for the detection of clinically important bacterium (*Staphylococcus aureus*) in air, nasal passage and skin samples [115]. It was noticed that the bacteria signals were significantly improved and several mass peaks from the nasal and air samples were identical with the standard *S. aureus* peaks. To improve the detection ability and sensitivity for bacteria species, titanium bacterial chip-based MALDI-MS was used as biosensor for the sensitive detection of pathogenic bacteria such as *Staphylococcus aureus subsp. aureus* and *Pseudomonas aeruginosa* [116]. Using this approach, bacteria was detected at <10^3^ cfu/mL. Our group also described the enrichment of peptides and proteins using ZrO_2_ and ZrO_2_-SiO_2_ NPs [117] and bare TiO_2_, TiO_2_-dopamine and TiO_2_-CdS [118] by MALDI-MS. The target analytes were effectively trapped and the concentrated target analytes (Leu-enk, Met-enk, thio peptide (thio) and angiotensin I and II (Angio-I and -II) and milk proteins) from the biocomplex samples through electrostatic [117] and covalent interactions [118] between nanomaterials and target analytes. These approaches provide efficient platforms for sensitive analysis of peptides and proteins (5–11 folds). Using this approach [117], several milk proteins were selectively isolated/concentrated from non-fat milk and the mass peaks at *m/z* 9,430, 11,489, 11,710, 14,173, 18,160, 18,975, 20,051, 23,574, 23,982 and 25,178 corresponded to proteose peptone PP81, γ_3_-casein, γ_2_-casein, α-lactoalbumin, β-lactoglobulin, κ-casein, γ_1_-casein, α_s1_-casein, β-casein, and α_s2_-casein, respectively were detected. Very recently, we described the dual application of 12-hydroxy octadecanoic acid (HOA)-modified barium titanate nanoparticles (BaTiO_3_ NPs) in MALDI-MS for the analysis of phospholipids (PLs; L-A-phosphatidyl-l-serine (PS) and L-A-phsophatidic acid sodium (PA)) and hydrophobic proteins in *E. coli* [119]. This method showed good linearity in the concentration ranges of 1.0–5.0 µM and 1.0–10.0 µM for PS and PA, respectively. The HOA-modified BaTiO_3_ NPs-assisted LLME coupled with MALDI-MS was successfully detected several hydrophobic proteins (membrane proteins—*ecnB* (P56549), *lpp* (P69776), and *osmE* (P23933); hypothetical membrane proteins—*yifL* (P39166) and *ygdI* (P65292); acetyl–acyl carrier protein (*ydhI*; *acetyl-ACP*, P0A6A8) and lipoproteins (*ecnB*, *lpp*, *osmE*, *yifL*, *ygdI*) and water-insoluble ATPase proteolipid (at *m*/*z* 8282; atpL, P68699)) in *E. coli*. It proved as a simple sample pretreatment for efficient extraction, preconcentration of hydrophobic proteins in biological samples prior to MALDI-MS analysis. These approaches proved as an alternate method to use sample cleanup method, which is promising either selective extraction of the desired analytes or selective rejection of interfering species. Table 2 shows the overview of semiconductor nanomaterials-based MALDI-MS for the biomolecules assays. Therefore, functionalized semiconductor nanomaterials-based MALDI-MS approaches are promising platforms for the efficient extraction and enrichment of trace level target species in biocomplex samples.

**Table 2:** Overview of semiconductor nanomaterials-based MALDI-MS for biomolecules assays.

Name of semiconductor NPs	Capping ligand	Analytes	Size (nm)	Detection limit	Technique	Reference
ZnS	MPA	Insulin, ubiquitin	-	85–91 nM	MALDI-MS	[96]
ZnS	N_3_	Milk and ubiquitin-like proteins	15	-	MALDI-MS	[97]
CdS	MPA	Peptides and proteins	5	-	LDI-MS	[98]
CdS	MPA	Digested proteins	5	-	ESI-MS	[100]
CdS	ATP, MUA	Peptides and proteins	15–30	0.01–63 nM	MALDI-MS	[101]
CdSe	MUA	Peptides and proteins	<10	-	LDI-MS	[102]
Cd^2+^-doped CNTs- CdS NPs	-	Cytochrome c and lysozyme	-	1–7 nM	AP-MALDI-MS and MALDI-MS	[103]
ZnSe	MPA	Leu-enk, Met-enk, HW6, substance P and Angio-II, and proteins (cytochrome c, myoglobin and lysozyme)	<5	-	MALDI-MS	[104]
ZnSe	MPA	Insulin, ubiquitin, cytochrome c, myoglobin and lysozyme	<10	-	MALDI-MS	[105]
Mn^2+^-ZnS	Cysteine	Peptides and proteins	5.1	~1.0 pM	MALDI MS	[91]
HgTe	MPA	Angio- I, Insulin, cytochrome c, BSA, IgG and *E. coli*	20	0.2–450 nM	SALDI- and MALDI-MS	[90]
HgTe	MPA	α_1_-antitrypsin−trypsin and IgG−protein G complexes,	-	0.5–3.0 µM	MALDI-MS	[106]
CdS:Mn/ZnS	DDTC	Tryptic digests of cytochrome c, lysozyme and BSA	6 ± 2	-	MALDI-MS	[107]
BaTiO_3_	-	Tryptic digests of α- and β- casein and milk proteins	30	-	MALDI-MS	[108]
SnO_2_ and TiO_2_	PMMA	Myoglobin	2–8	1 nM	SALDI-MS	[109]
Au, TiO_2_, Se, CdTe QDs, Fe_3_O_4_, and Pt	-	Glutathione, Angio-I, insulin, cytochrome c and chymotrypsin	-	140–4400 fM	SALDI-MS	[110]
Mg(OH)_2_	Oleic acid	Gramicidin D, valinomycin, *E. coli*	<35	-	MALDI-MS	[111]
ZnO	-	*E. coli*	-	-	MALDI-MS	[112]
ZnO	-	*Staphylococcus aureus*	-	-	MALDI-MS	[113]
CdS	MPA	Extracellular polysaccharides in *E. coli*	<5	-	MALDI-MS	[114]
TiO_2_	-	*Staphylococcus aureus*	-	-	MALDI-MS	[115]
TiO_2_	bacteria	*Staphylococcus aureus subsp. aureus* and *Pseudomonas aeruginosa*	-	-	MALDI-MS	[116]
ZrO_2_ and ZrO_2_-SiO_2_	-	Leu-enk, Met-enk, HW6, and milk proteins	20–30	75–105 fM	AP-MALDI-MS	[117]
TiO_2_-dopamine and TiO_2_-CdS	-	Gramicidin D, myoglobin, cytochrome c, α- and β-caseins	5–20	1 nM	MALDI-MS	[118]
BaTiO_3_	HOA	PLs and Hydrophobic proteins in *E. coli*	30–40	0.20–0.40 µM	MALDI-MS	[119]

## 5. Conclusions and Future Perspectives

The possibility to control and tune the unique optical and electronic properties of semiconductor NMs through their dimensions paves the way to the application of nanomaterials-based fluorescence spectrometric and MALDI-MS as versatile analytical tools. The need for ultrasensitive bioassays and the trend towards miniaturized assays have made the semiconductor nanomaterials-based fluorescence spectroscopy and MALDI-MS approaches to be the most popular field in proteomics research. A wide variety of semiconductor nanomaterials have been introduced into fluorescence spectroscopy and MALDI-MS for sensitive identification of proteins from various biological samples. Semiconductor nanomaterials can be used as optical probes and affinity/concentrating probes to enhance/decrease fluorescent signals in fluorescence spectroscopy and to enrich/enhance trace level proteins signals in MALDI-MS. The functionalized semiconductors QDs have showed high ability to quench/enhance or trap/concentrate the target analytes in fluorescence spectroscopy and MALDI-MS. These approaches are based on the dipole-dipole interaction in fluorescence and covalent or hydrophobic or electrostatic interactions in MALDI-MS. On the basis of the current state of art in nanomaterials-based bioanalytical tools, semiconductor nanomaterials-based fluorescence spectroscopy and MALDI-MS approaches are efficient tools for rapid and sensitive identification of biomolecules in modern proteomic research.

For QDs-based fluorescence approaches based on the FRET principle, tuning electronic property of QDs by controllable modification and organic framework on QDs surfaces, and development of techniques to integrate QDs into practical devices having high sensitivity, selectivity with acceptable reproducibility, reliability, non-toxic and low cost remains a big challenge. As for QD-based fluorescent probes, although QDs have distinct advantages over organic dyes such as high fluorescent ability, biocompatibility, facile functionalization and extremely bright (40%–80% room temperature quantum efficiency) emission tunable to any desired wavelength in the spectral range of 500–800 nm. The use of organic framework on QDs surfaces not only permits for efficient FRET but also allows for a purposeful design of a wide variety of organic (dyes, conjugated polymers) and strong interactions with target analytes, which enhances the optical and conducting properties of QDs with robustness and flexibility. Therefore, surface chemistry should be designed taking into consideration the chemical nature of the semiconductor QDs and at the same time affording efficient ligand coupling and providing optimal ligand presentation. Furthermore, there is a great demand for effective surface coupling chemistry for controlled coupling with bioactive species to enable specific targeting in cellular and *in vivo* imaging of biomedical samples. The organic framework on QDs surfaces is likely to continue, which should be highly beneficial to the overall development and mechanistic understanding of semiconductor QDs as fluorescent probes for biomolecule assays as more competitive alternatives to conventional organic dyes.

The supramolecular chemistry of QDs coupled MALDI-MS provides an excellent reproducibility, high mass resolution, allows quantitative determination of concentrations of a wide variety of biomolecules (peptides and large proteins > 1.0 kDa). Semiconductor QDs-based MALDI approaches proved as a powerful bioanalytical tools, providing the advantages of multifunctional roles (as heat absorbers and as affinity probes) and ease of sample preparation for proteomics. To this end, development of suitable chemical routes and organic framework approaches for precise control over size, size distribution, morphology and active reacting groups of QDs is urgently needed, as this is closely correlated to the performance of the QDs-based MALDI-MS approaches for biomolecules assays and for the safety issues as well. Although these QDs-based MALDI-MS proved a powerful bioanalytical tool for proteomics studies, much more work remains to be done for the applications of QDs in MALDI-MS and MALDI imaging mass spectrometry to enable specific targeting in cellular and *in vivo* imaging and related biomedical applications.
